# The Longitudinal Implementation Strategy Tracking System (LISTS): feasibility, usability, and pilot testing of a novel method

**DOI:** 10.1186/s43058-023-00529-w

**Published:** 2023-11-28

**Authors:** Justin D. Smith, Wynne E. Norton, Sandra A. Mitchell, Christine Cronin, Michael J. Hassett, Jennifer L. Ridgeway, Sofia F. Garcia, Raymond U. Osarogiagbon, Don S. Dizon, Jessica D. Austin, Whitney Battestilli, Joshua E. Richardson, Nathan K. Tesch, David Cella, Andrea L. Cheville, Lisa D. DiMartino, the IMPACT Consortium

**Affiliations:** 1 Department of Population Health Sciences, School of Medicine, University of Utah, Spencer Fox Eccles, Salt Lake City, UT USA; 2 Departments of Psychiatry and Behavioral Science and Medical Social Sciences, Northwestern University Feinberg School of Medicine, Chicago, IL USA; 3 Division of Cancer Control and Population Sciences, National Cancer Institute, Bethesda, MD USA; 4 Division of Population Sciences, Dana-Farber Cancer Institute, Boston, MA USA; 5 Departments of Medical Oncology and Quality & Patient Safety, Dana-Farber Cancer Institute, Boston, MA 02215 USA; 6 Robert D. and Patricia E. Kern Center for the Science of Health Care Delivery and Division of Health Care Delivery Research, Mayo Clinic, Rochester, MN USA; 7 Departments of Psychiatry and Behavioral Science and Medical Social Sciences, Northwestern University Feinberg School of Medicine, Chicago, IL USA; 8 Multidisciplinary Thoracic Oncology Program, Thoracic Oncology Research Group, Baptist Cancer Center, Memphis, TN USA; 9 Division of Hematology-Oncology, Department of Medicine, Legoretta Cancer Center, The Warren Alpert Medical School of Brown University, and Lifespan Cancer Institute, Providence, USA; 10 Division of Epidemiology, Department of Quantitative Health Sciences, Mayo Clinic, Scottsdale, AZ USA; 11 Center for Clinical Research Informatics, RTI International, Durham, NC USA; 12 Center for Health Informatics, RTI International, Research Triangle Park, Fayetteville, NC USA; 13 Robert D. and Patricia E. Kern Center for the Science of Health Care Delivery, Mayo Clinic, Rochester, MN USA; 14 Department of Medical Social Sciences, Northwestern University Feinberg School of Medicine and Robert H. Lurie Comprehensive Cancer Center, Northwestern University, Chicago, IL USA; 15 Department of Physical Medicine and Rehabilitation, Mayo Clinic, Rochester, MN USA; 16 Peter O’Donnell Jr. School of Public Health, University of Texas Southwestern Medical Center, Dallas, TX USA

**Keywords:** Common data elements, Implementation strategies, Modifications, Reporting, Methodology, Specification

## Abstract

**Background:**

Systematic approaches are needed to accurately characterize the dynamic use of implementation strategies and how they change over time. We describe the development and preliminary evaluation of the Longitudinal Implementation Strategy Tracking System (LISTS), a novel methodology to document and characterize implementation strategies use over time.

**Methods:**

The development and initial evaluation of the LISTS method was conducted within the *I*mproving the *M*anagement of Sym*P*toms during *A*nd following *C*ancer *T*reatment (IMPACT) Research Consortium (supported by funding provided through the NCI Cancer Moonshot^SM^). The IMPACT Consortium includes a coordinating center and three hybrid effectiveness-implementation studies testing routine symptom surveillance and integration of symptom management interventions in ambulatory oncology care settings. LISTS was created to increase the precision and reliability of dynamic changes in implementation strategy use over time. It includes three components: (1) a strategy assessment, (2) a data capture platform, and (3) a User’s Guide. An iterative process between implementation researchers and practitioners was used to develop, pilot test, and refine the LISTS method prior to evaluating its use in three stepped-wedge trials within the IMPACT Consortium. The LISTS method was used with research and practice teams for approximately 12 months and subsequently we evaluated its feasibility, acceptability, and usability using established instruments and novel questions developed specifically for this study.

**Results:**

Initial evaluation of LISTS indicates that it is a feasible and acceptable method, with content validity, for characterizing and tracking the use of implementation strategies over time. Users of LISTS highlighted several opportunities for improving the method for use in future and more diverse implementation studies.

**Conclusions:**

The LISTS method was developed collaboratively between researchers and practitioners to fill a research gap in systematically tracking implementation strategy use and modifications in research studies and other implementation efforts. Preliminary feedback from LISTS users indicate it is feasible and usable. Potential future developments include additional features, fewer data elements, and interoperability with alternative data entry platforms. LISTS offers a systematic method that encourages the use of common data elements to support data analysis across sites and synthesis across studies. Future research is needed to further adapt, refine, and evaluate the LISTS method in studies with employ diverse study designs and address varying delivery settings, health conditions, and intervention types.

**Supplementary Information:**

The online version contains supplementary material available at 10.1186/s43058-023-00529-w.

Contributions to the literature

•Building on implementation strategy specification and reporting standards, LISTS advances precision and reproducibility in capturing the use and modification of implementation strategies over time
•LISTS provides the field with a feasible and usable approach for characterizing and reporting variations in strategy use and documenting how and why strategies are modified over time within and across studies.
•A systematic methodology such as LISTS is critical for capturing data that can be synthesized across multiple sites and multiple studies. The LISTS method supplies common data elements and supports integrated data analysis across multiple studies.

## Background

Characterizing, tracking, and reporting implementation strategies over time is critical for advancing the science. Several methods for tracking implementation strategies have been proposed recently, underscoring the importance of continued development of methods to accurately assess and monitor what strategies are employed, and how and why they change over time during implementation studies. Given the strengths and limitations of each of these approaches (see Table 1), continued methodologic work is needed to test and optimize a methodology and a data capture interface that balances rigor, feasibility, and usability. The novel Longitudinal Implementation Strategy Tracking System (LISTS) method described in this article was developed to address the limitations of the existing methods briefly reviewed in the following section and to advance the science of strategy tracking toward greater transparency and use of common data elements.

**Table 1:** Summary of other implementation strategy tracking methods

Authors	Relevant Framework	Methods	Strengths	Limitations
Bunger et al. [1]	• Expert recommendations for Implementing Change (ERIC)	• Activity logs coded by research staff	• Low cost to the implementers	• Time intensive coding required • Limited strategy specifications collected
Boyd, Powell, Endicott, and Lewis [2]	• Powell et al. [3] compilation from 2011	• Coded team meeting recordings	• Leveraging existing implementation team meetings reduced burden • Captured change over time	• Meetings were not structured to obtain all necessary information about specific strategies • Completeness of coded strategies is unknown
Rabin et al. [4]	• Stirman et al.’s [5] expanded Framework for Reporting Adaptations and Modifications to Evidence-based interventions (FRAME) • Components of RE-AIM	• Real-time data collection via an adaptations worksheet • Semi-structured interviews at 6-month timepoints	• Provided flexibility • Low burden on implementers	• Time and training of research staff for administration and coding • FRAME not tailored to implementation strategies • Lag-time from semi-structured interviews may have led to retrospective error
Rabin et al. [4]	• Modified FRAME • Consolidated Framework for Implementation Research (CFIR)	• Tracked modifications to an a priori strategy protocol	• Results in fairly granular and comprehensive data	• Time and resource burden on the study team • The time (dose) involved in each strategy was not captured • Implementers themselves were not involved in the tracking or coding
Walsh-Bailey et al. [6]	• None stated	• Brainstorming log (*low* structure) • Activity logs (*moderate*) • Detailed tracking logs (*high*) • Random assignment to implementation strategies	• Activity log method deemed most feasible of the three methods	• Validity and necessary detail provided by each method, balanced on burden and perceptions, was not assessed • Limited number of implementers engaged in evaluation • Intervention evaluated was relatively simple (results not generalizable)

### Brief review of existing implementation strategy tracking methods

Bunger et al. [1] used activity logs, completed by implementers, that captured the purpose (to identify the type of strategy), estimated length of time (to estimate dosage), and individuals involved (to specify actors) in implementation studies. The strategies were later coded by research staff to name the strategy according to the Expert Recommendations for Implementing Change (ERIC) taxonomy [7]. While this approach was low cost, coding by research staff was required, and not all aspects of strategy specification were collected.

 Boyd, Powell, Endicott, and Lewis [2] proposed an approach that coded implementation team meeting recordings for reporting and specification elements [8] and categorized the strategies according to the Powell et al. [3] compilation from 2011. While the use of existing implementation team meetings reduced burden, the meetings were not structured to obtain all necessary information about specific strategies, and the completeness of the strategies ultimately coded is unknown. The tri-weekly schedule represented a strength in terms of capturing change over time, but this was not an explicit goal of the project, or the coding scheme applied.

Rabin et al. [4] used a multimethod, multilevel assessment approach to capture adaptations to implementation strategies across a multi-site study with four sites. Data collection methods included an adaptations worksheet, for use in real time, based on Stirman et al.’s [5] expanded Framework for Reporting Adaptations and Modifications to Evidence-based interventions (FRAME), with additional components from the Reach, Effectiveness, Adoption, Implementation, and Maintenance (RE-AIM) framework [9]. Semi-structured interviews were also conducted at two time-points 6 months apart. This approach offered flexibility and low burden on the implementers but required time and training of research staff for administration and coding. Limitations included the use of FRAME, which is designed to characterize the fidelity of delivering evidence-based interventions and not tailored to implementation strategies. In addition, the time-lag between deployment of implementation strategies and the semi-structured interviews may have contributed to recall bias, although real-time tracking logs were sometimes used to prompt interview participants’ recall.

In another study, the research team tracked modifications to an a priori strategy protocol using a modified version of the FRAME applied to weekly implementation team meeting notes [10]. The Consolidated Framework for Implementation Research (CFIR) [11] was used to code contextual factors or barriers discussed during the meetings as they related to strategy changes, and explicitly asked about the addition of strategies not prescribed in the study protocol. Challenges with this approach included the time and resource burden on the study team, the time (dose) involved in delivering each strategy was not captured and that the implementers themselves did not participate in either data capture or coding.

Walsh-Bailey et al. [6] provide the only study, to our knowledge, comparing different methods for strategy tracking. The data collection approaches varied with respect to their degree of structure: brainstorming log (*low* structure), activity logs (*moderate*), and detailed tracking logs (*high*). The intervention developer, implementation practitioners, and mental health professionals were randomly assigned to use one of the three methods each week to prospectively track implementation strategies and intervention adaptations. The activity log method was deemed most feasible. While this study captured the perspectives of those asked to provide data on strategy use and adaptations, the validity and precision provided by each method, balanced by burden and perceptions of usability, was not assessed. Additionally, only 11 implementation practitioners were engaged in the evaluation, and the intervention being tested was relatively straightforward to implement compared to other research studies in which strategy tracking methods have been assessed.

Other studies have tracked strategy use at a very high or broad level [12]. While such methods offer ease of completion, they are unlikely to provide the necessary details to distinguish among related strategies or to understand the ways in which strategies were modified. Moreover, such high-level approaches limit the opportunity to understand why strategies were or were not effective and reduce the ability to synthesize the data with other studies.

**Fig. 1:**
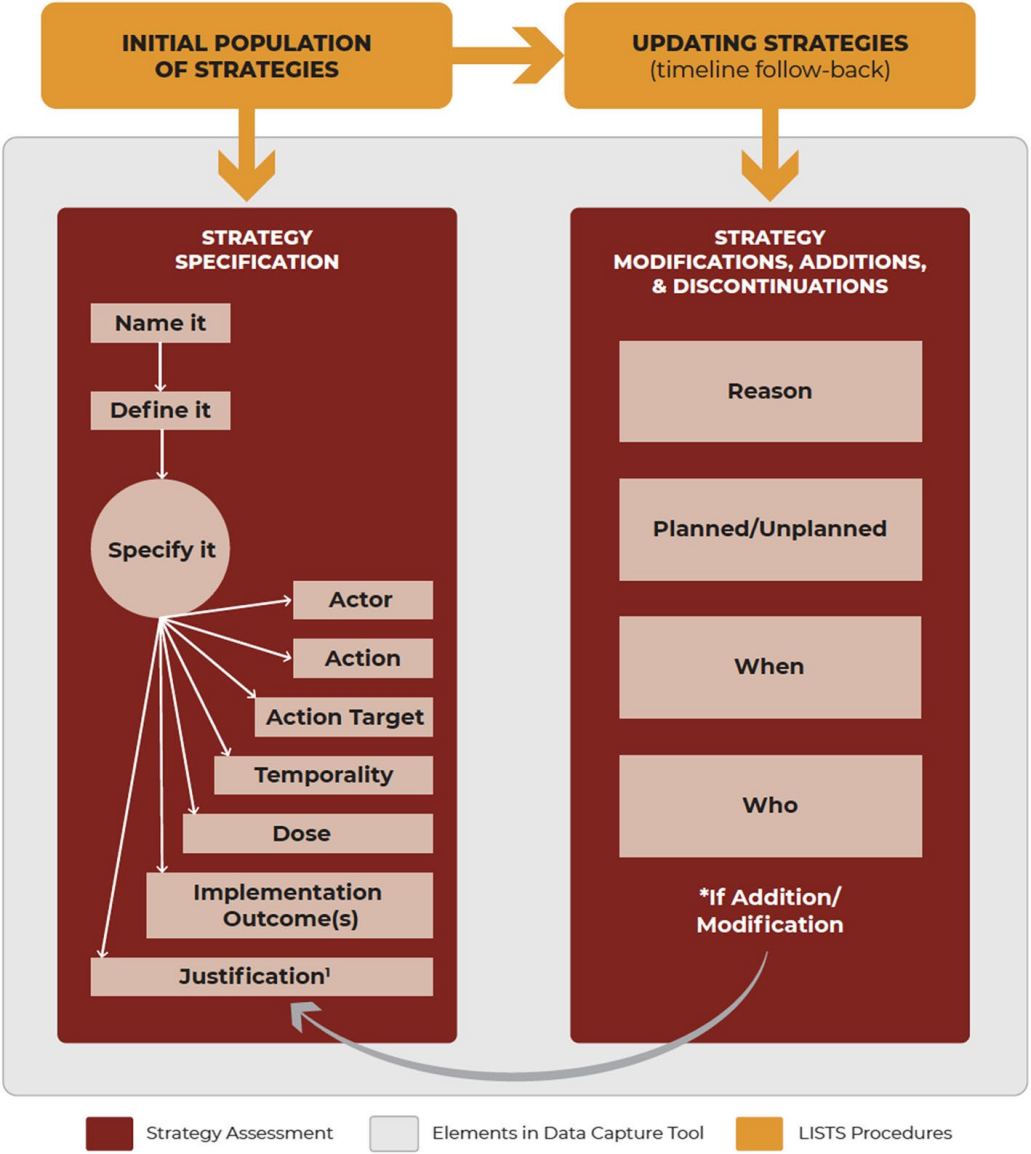
LISTS Strategy Assessment, Elements in Data Capture Platform, and Procedures *Notes*. ^1^ LISTS includes the list of CFIR constructs to select. Additional text is necessary for providing a complete justification for each strategy. LISTS procedures are described in the User’s Guide

While all of these recently developed strategy tracking systems have strengths, they also have notable limitations related to measurement timing and frequency (either too infrequent to validly capture dynamic change or bordering on being too intensive and thus infeasible) and inconsistent adherence to reporting guidelines, which are a product of the data source or data collection method. Building on recent calls for advancing the science of implementation strategies [13–15], and to address some limitations of existing strategy tracking systems, we developed the Longitudinal Implementation Strategies Tracking System (LISTS) method, a systematic methodology for assessing, documenting, and tracking strategy use over time. LISTS includes three components: (1) a strategy assessment, 2) a data capture platform, and 3) a User’s Guide describing the procedures (see Fig. 1). Below, we detail the development of the LISTS method and describe how it was used in three hybrid effectiveness-implementation trials [16–18]. We then provide data on the initial feasibility, acceptability, and usability of LISTS from a survey completed by implementation researcher and practitioner teams who had used LISTS within each of their respective studies for approximately 12 months.

### Development of the LISTS method

The development and initial testing of LISTS occurred within the *I*mproving the *M*anagement of Sym*P*toms during *A*nd following *C*ancer *T*reatment (IMPACT) Consortium supported by funding provided through the Cancer Moonshot^SM^. The goal of the IMPACT Consortium is to support the rigorous development, implementation, evaluation, and scalability of electronic health record (EHR)-integrated symptom surveillance and management systems in ambulatory oncology [19]. These systems also provide self-management support to patients and clinical decision support to clinicians to manage symptoms in a manner that is consistent with evidence-based guidelines. The IMPACT Consortium is comprised of three major components: 1) three individual Research Centers (RCs), each conducting hybrid effectiveness-implementation trials testing routine symptom surveillance and evidence-based symptom management interventions in ambulatory oncology care settings; 2) a Coordinating Center; and (3) NCI program staff who participate as project scientists. Several content- or topic-specific workgroups are also part of the Consortium, including the Implementation Science Workgroup (ISWG), which includes representatives from all three RCs, the Coordinating Center, and NCI program staff [19].

The ISWG conceived the LISTS method to achieve the Consortium goal of common data elements, data synthesis, and analyses of implementation strategies both within and between the three hybrid design studies. As each RC is using a variation of a cluster randomized stepped-wedge trial design [16–18], the ISWG also recognized the need for detailed strategy reporting and tracking to support the interpretation of trial outcomes within and across the RCs. This was particularly important given the complexity of the trials, which involved multicomponent implementation strategies and complex interventions in multiple large healthcare delivery systems.

### Components of the LISTS method

The LISTS method includes three components: (1) a strategy assessment, (2) a data capture platform, and (3) a User’s Guide of procedures for its use, as detailed below.

### Strategy assessment

Strategy assessment included strategy specification, reporting, and modification, the elements of which were drawn from multiple sources. For strategy specification and reporting, we followed recommendations by Proctor, Powell, and McMillen [8]. These include *naming* (using language consistent with the existing literature) and *defining* (operational definitions of the strategy and its discrete components) the strategy and specifying the *actor* (who enacts the strategy), *action* (active verb statements concerning the specific activities, steps, or processes), *action targets* (the strategy’s intended target according to a conceptual model or theory), *temporality* (duration of use and frequency/interval or indication for use), *dose* (how long it takes to deliver the strategy each time), *implementation outcome(s)* likely to be affected, and the (empirical, pragmatic, or theoretical) *justification* for use.

To standardize the naming and defining of strategies using phrasing and conceptualizations consistent with the literature, the data capture platform (described next) was pre-populated with the 73 discrete strategies from the ERIC compilation [7]. We used the Proctor et al. [20] taxonomy of implementation outcomes to provide standardized definitions for acceptability, adoption, appropriateness, cost, feasibility, fidelity, penetration/reach, and sustainability/sustainment. Auto-population of the CFIR constructs was used for the justification element. An accompanying narrative justification could also be used to supplement LISTS data on determinants (from CFIR) that the strategy was selected to address. While the LISTS method can be used with other taxonomies and frameworks, we selected these because of their familiarity and widespread use in the field.

To capture strategy modifications (which includes changes to strategies as well as strategy additions and discontinuations), we incorporated four elements from the Framework for Reporting Adaptations and Modifications Expanded to Evidence-based Implementation Strategies (FRAME-IS) [21]. These included the *When*, *Who*, how *Widespread* (one response option), and *Planned/Unplanned* elements. The additional elements of FRAME-IS were thought to be better suited for narrative or free-text responses (e.g., *Nature* of the modification, the *Goal*), and thus were not included in the REDCap. These elements can be included in an accompanying document if desired by research and implementation teams.

Consistent with FRAME-IS, strategies entered in the data capture platform (described in the next section) can be updated to indicate modifications to a strategy. For such modifications (including discontinuation), branching logic within the LISTS data capture interface prompts users to document the *reason* for the strategy change (e.g., ineffective, infeasible), whether the strategy change was *planned* (e.g., part of an a priori protocol) or *unplanned* (e.g., response to emergent implementation barrier), and *who* was involved in the strategy change decision (e.g., leadership, research team, clinicians). When a new strategy is added, the same “was it planned or unplanned” and “who was involved” questions are prompted, along with the reason for deploying the new/additional strategies (with response options of “to address an emergent barrier” or “to complement/supplement other strategies to increase effectiveness”). Data elements for specifying and reporting strategies, as described above, are also prompted.

#### Data capture platform

The data capture platform was programmed in Research Electronic Data Capture (REDCap) [22], a secure web application for creating and managing online surveys and databases. Screenshots of the REDCap module are included in Supplemental File 1 (SF1) as Supplemental Figs. 1–5 (labeled as SF1 Figure X in the text). The data capture platform was beta tested by the ISWG prior to finalization and use by the RCs in their respective studies. We selected REDCap because it was used by all the RCs in the consortium (e.g., for survey administration) and was familiar to the research teams. LISTS includes branching logic and auto-population of implementation determinants per CFIR (SF1 Fig. 1), Proctor et al. implementation outcomes (SF1 Fig. 2), and other response options described below (e.g., *Planned/Unplanned* elements, SF1 Fig. 2).

Two features of the LISTS data capture interface are important to highlight since they improve precision and data interpretability across diverse contexts. First, users defined a single level, often associated with study units within their studies (SF1 Fig. 4). For example, RCs could select to specify clusters (comprised of smaller clinical units), health systems, or other meaningful units within their cluster randomized design. The IMPACT studies had diverse unit compositions consistent with their respective study designs that were specified as the levels in LISTS. For example, units in one study were entire health systems whereas others were clusters of specific locations of care (i.e., ambulatory oncology practices). For all three of the IMPACT studies, strategy tracking occurred at the cluster level, as it was expected that the centralized implementation approach would result in more variation in implementation strategies at the cluster level rather than at the clinic level. Each time a strategy is added or modified, the user has the option to specify in REDCap whether it applies to “all units” or only “specific individual units” (and designate all that apply). This feature is essential to capture the level of specificity needed to interpret the data within and across studies, particularly in large, multi-site trials conducted in multiple health systems, where the participating components may have very different implementation challenges, needs, and barriers. Importantly, this feature allows for heterogeneity to be captured, and offers both precision and flexibility in using the LISTS method in future research studies with diverse settings and contexts.

Second, the dashboard function in REDCap was programmed to provide users with a snapshot of the strategies entered. The dashboard is color-coded (SF1 Fig. 5) to indicate whether the strategy is active or has been discontinued, and whether data entry about that strategy is incomplete (i.e., some data fields are not entered). This user-friendly dashboard facilitates efficient and timely review of implementation strategies thereby supporting teams consistent tracking of strategy use and modification.

### User’s Guide

Members of the ISWG (JDS, WEN, and LDD) prepared an initial draft of a User’s Guide for the LISTS method for RCs. In developing the guide, frequency of using LISTS was a primary consideration, with the goal of balancing rigor with burden. Given considerations for stage of implementation, complexity of implementation, and desire to incorporate LISTS into ongoing project activities (e.g., monthly meetings with implementation teams, which included implementation researchers and implementation practitioners), we suggested using a timeline follow-back procedure [23] to facilitate accuracy during data entry. Timeline follow-back procedures involve ongoing retrospective reporting over relatively short periods to minimize retrospective error and ensure that dynamic changes are captured. RCs were encouraged to enter data at least quarterly, with the option for more frequent entry (e.g., monthly, twice-monthly, or bi-monthly). Features that allowed for this more frequent data entry were important to include given that modifications to strategies may be more likely to occur during earlier stages of implementation compared to later stages [24]. Such flexibility in the timing and frequency of data entry also allows for the capture of changes that may occur due to unexpected disruptions to the service context, such as policy changes or a global pandemic [23].

The draft LISTS User’s Guide was provided to each RC. Teams were encouraged to adapt the procedures in the User’s Guide to meet their needs, and modifications were incorporated into LISTS procedures moving forward. Confirmation of responses in LISTS, and inquiries for specific strategy data to implementers in the healthcare system, were also encouraged. Questions that arose as teams used the LISTS method were shared and discussed with the ISWG and reported back to the implementation teams. Examples included clarification of procedures and questions about how best to answer certain strategy specification elements. The LISTS User’s Guide and REDCap module installation instructions and files are available at: https://github.com/uofu-ccts/LISTS_REDCap_project.

## Methods

### Initial evaluation of the feasibility, acceptability, and usability of the LISTS method

#### Context

The LISTS method was assessed in each of the three RCs that are part of the IMPACT Consortium. Briefly, each RC focuses on improving symptoms management during and after treatment among patients with solid tumor cancers receiving systemic therapy in academic health centers. Two of the three RCs conducted their trial in one health system that included multiple clinic locations (one had 32 outpatient medical oncology clinics in a large metropolitan area [18], the other had 15 care sites across three states [17]). The third RC conducted their trial in six healthcare systems across nine states each with at least one primary cancer center and some with multiple hospitals and clinics [16]. Additional details about the IMPACT Consortium can be found in Wilder-Smith et al. [19]. Details about each of the three trials from each RC can be found in their respective protocol papers [16–18].

### Participants

Each RC included a small team of researchers, research staff, and implementers who were primarily responsible for using the LISTS method. The roles included principal investigator/co-investigators, project coordinator, project manager, physician-scientist, quality lead, and operations partners. Each team was asked to complete one survey, reflecting on their experiences in using the LISTS method over a 12-month period. Since use of LISTS was a team-based approach, rather than being completed by a single researcher or practitioner, teams (vs. individuals) were asked to review, discuss, and reach consensus on responses to the survey. Thus, although data presented herein are only results from three surveys (*n* = 3; one from each RC), they include the collective feedback of teams within each RC.

### Survey

A brief survey was developed to evaluate the LISTS method by users. The survey included questions to assess the feasibility, acceptability, and usability of the method; to rate specific data elements in LISTS; to understand and describe variations in how LISTS was used relative to the procedures specified in the User’s Guide; and to collect suggestions for future improvements. A copy of the survey is provided in Supplemental File 2.

### Strategy assessment

With respect to feasibility, acceptability, and usability, each RC was asked to rate the difficulty of assessing each of 11 aspects of strategy assessment (e.g., *Selecting a specific strategy*, *Frequency of strategy use*) on a 5-point Likert scale (1 = Very Easy, 2 = Easy, 3 = Neutral [neither difficult nor easy], 4 = Difficult, 5 = Very Difficult).

### Data capture platform

Ten items from a modified version of the System Usability Scale (SUS) [25] were used to assess the usability of the REDCap data capture platform developed for LISTS. Examples include, “I thought the LISTS REDCap system was easy to use” and “I found the LISTS REDCap system very cumbersome/awkward to use,” all rated on a 5-point Likert scale (1 = Strongly Disagree, 2 = Disagree, 3 = Neither Agree nor Disagree, 4 = Agree, 5 = Strongly Agree).

### User’s Guide

Several questions assessed the procedures used by each RC to use the LISTS method, including any variations to the procedures initially defined in the User’s Guide. Specifically, each RC was asked to record the dates they met regarding LISTS and indicate whether the meeting was used for *initially populating the* strategy (entering strategies in use/strategies used and stopped) or for *updating* the strategy (reporting strategy modifications, strategy discontinuation, etc.). Each RC was asked to describe the roles (e.g., project coordinator, implementation scientist, quality improvement lead, physician, nurse manager) of the team members who used LISTS, and indicate whether each person was involved in meetings routinely (i.e., majority of meetings) or occasionally (i.e., minority of meetings). RCs were asked to briefly describe the process they used for *initially* populating strategies and for *updating* strategies over time. RCs were also asked to list the approaches used to complement and/or confirm the accuracy of data entries (options provided: review of meeting notes/agendas, review of calendar entries, checking with on-the-ground staff/implementers) and indicate frequency of use of each approach (1 = Rarely [once or twice], 2 = Occasionally [a few times], 3 = Frequently [many times], 4 = Always [nearly every strategy]).

### Data analysis

A mean difficulty score was computed from the Likert-type scale responses for each of the 11 LISTS strategy assessment data elements (see Table 2). To evaluate the LISTS data capture platform, the SUS scores were calculated using an online scoring program consistent with developer scoring guidelines and interpreted according to the percentile ranks, grades, and descriptions provided by Suaro [26]. The estimated time involved in both initially populating the strategy and updating the strategy in LISTS was totaled within each RC. The role and procedure items were summarized and interpreted descriptively.

**Table 2:** Survey results for implementation strategy assessment data elements

Question: Rate the difficulty of assessing each of the following elements of LISTS	Mean	Point spread (range)
Location of strategy use	1.33	1
Selecting a strategy category (from ERIC compilation)	1.67	1
Actor(s) who used the strategy	1.67	1
Reason for strategy stoppage	2.33	1
Prospective vs. not	2.33	2
Implementation outcome target	2.67	1
Why was strategy used (i.e., selection of barriers strategy thought to be addressing)	2.67	3
Individuals involved in the strategy	2.67	2
Selecting a specific strategy (from ERIC compilation)	2.67	3
Frequency of strategy use	3.33	3
How long does it take to do the strategy (dose)	4.33	1

5-point Likert scale (1 = Very Easy, 2 = Easy, 3 = Neutral [neither difficult nor easy], 4 = Difficult, 5 = Very Difficult)

## Results

Results from the three surveys are presented collectively to mask the identity of any individual RC and its members.

### Strategy assessment

Aspects of the implementation strategy assessment that were deemed “very easy” or “easy,” all with good agreement (1-point spread), were location of strategy use (study units), identifying the actor (person(s) who enact the strategy), and selecting the strategy category from the ERIC compilation (see Table 2). Selecting the discrete strategy within a category was rated as more difficult by some centers. The two most difficult aspects of the strategy assessment related to the frequency of use (e.g., every patient encounter, weekly; 3-point spread) and the dose (how long does it take to do the strategy; 1-point spread). While not captured in the survey itself, authors from the RCs suggested that this was in part due to this information not being routinely documented in meeting notes or other sources; thus, obtaining estimates for these data elements required asking the implementers (Actors) of the strategy. Difficulty obtaining the data for these two elements should not be misconstrued as the data being unreliable or being of low validity. The data simply required more time and effort to obtain. Despite these two items standing out as difficult, the remaining items had mean score range of 1.33–2.67, which can be interpreted as easy to neutral (neither easy nor difficult).

### Usability of the data capture platform

With respect to the data capture platform, REDCap, the mean score of the SUS across the three RCs was 67.5 or a percentile rank of 49th percentile. Consistent with the established interpretation of SUS scores [25], this score reflects a “C” grade, suggesting that the REDCap platform for capturing implementation strategy data is relatively usable but would benefit from improvement.

### Feasibility of LISTS procedures

The team composition for using the LISTS method varied to some degree at each RC, but all included implementation researchers. Co-investigators and other project staff with knowledge of the implementation strategies used within each RC were involved in meetings specific to LISTS. Implementers not involved in the meetings were consulted regarding LISTS data elements, as needed.

The time involved in both *initially populating the* strategy and *updating* the strategy varied by RC. Two of the three RCs are conducting their studies within a single healthcare system, while the third involves six distinct health systems with a centralized research team and coordinating center structure. RCs using a single healthcare system spent an average of 8 h distributed across 8 meetings each in the *initial* strategy population of LISTS. However, they varied widely in time spent *updating* strategy population in LISTS: one site spent a total of 12 h distributed across 7 meetings while the other RC spent a total of 1 h distributed across 3 meetings. The multi-system RC integrated LISTS initial strategy and updated strategy population into existing monthly implementation team meetings (6 meetings per month–one for each health system). They estimated spending a total of 21 h for initial population of strategies and a total of 42 h for updating strategies over the course of a year, respectively.

There were several common approaches across RCs for the *initial* strategy population of LISTS. Specifically, all RCs: (1) used the full list of ERIC strategy categories as a prompt for reporting strategies used; (2) entered data into an Excel spreadsheet (i.e., the back-end data form from LISTS exported from REDCap) and only entered the data into REDCap after data elements were verified and complete; (3) routinely confirmed LISTS data with other sources (e.g., other team members, calendars, meeting notes) as needed by strategy type and data element; (4) required team/unit/study leads to sign-off on the strategy prior to entry in REDCap; and (5) designated a single individual responsible for compiling and entering strategies into the Excel spreadsheet and subsequently into REDCap.

Inclusion of an RC whose study design involved multiple health systems highlighted an important additional step for strategy documentation and consolidation across study sites. Implementers from each of the separate systems documented strategies in a stand-alone REDCap database created by their consortium-specific evaluation team to allow for the capture of both site- and system-specific processes. These entries were then sent to the coordinating center’s central team for validation, harmonization, PI review and sign-off, and entry into the single LISTS REDCap entry for that RC.

Concerning procedures for *updating* the strategy in LISTS, all three RCs used routine check-ins with implementation practitioners regarding strategy modification. Two of the RCs reviewed the dashboard of active strategies in REDCap as a prompt for considering strategy modifications. One RC sent periodic emails to implementers to inquire about modifications. One RC conducted a formal review of all strategies (both those being used and whether they were modified from prior specification) as each study unit in the stepped-wedge trial moved from the control to the experimental condition.

RCs varied with respect to their frequency in using four data verification procedures from *Occasionally* to *Always*: reviewing meeting notes/agendas (most frequently used); reviewing calendars (occasional to frequent use); checking with on-the-ground implementers (occasional to always); and emails to PIs/Project Managers after LISTS-related meetings to confirm strategy specification and modifications (occasional use).

## Discussion

Tracking and reporting implementation strategies accurately, precisely, and comprehensively is a critical and necessary step for advancing the field of implementation science more broadly. The LISTS method builds on the processes, lessons learned, and noted limitations of the still relatively sparse reports of strategy tracking methods in the published literature [1, 2, 4, 10, 12]. Specifically, LISTS was developed to be responsive to all five areas noted by Powell et al. [13] and designed to facilitate the routine capture of detailed implementation strategy use and modification at regular intervals. The data elements in LISTS were derived from well-accepted frameworks and models in the literature and are captured in detail to facilitate cross-site and cross-study analyses of strategy use and modification. Importantly, LISTS was developed with an eye toward its potential use in future research. While developed within the specific context of the IMPACT Consortium, the LISTS data capture platform can be readily adapted and used in other research studies. For example, some may choose to use the existing REDCap software (available at https://github.com/uofu-ccts/LISTS_REDCap_project), while others may decide to use Excel or another software platform, such as R Shiny [27] and update the LISTS procedures accordingly.

The LISTS method demonstrated preliminary evidence of feasibility, acceptability, and usability, with some caveats. For example, users noted the need for implementation science expertise to complete some of the methods effectively. This was mainly due to understanding terminology in CFIR and ERIC, and the often-subtle differences between implementation determinants and strategies in the two taxonomies. It was also necessary for each of the three RCs to distinguish the components of the EHR-delivered symptom surveillance and management intervention from the implementation strategies. This ensured alignment and consistency across the RCs prior to initiation of the LISTS method. For example, creation of a clinical alert for severe symptoms was categorized as an implementation strategy across all RCs, while components related to the collection of symptom data were considered part of the intervention.

Some modifications to the procedures in the User’s Guide were also needed by the RCs to compile strategies across multiple sites. There is a need for prospective training and guidance for individuals who will participate in data collection, data cleaning, and validation procedures. The need for data cleaning and validation of strategy entries is essential for all studies, but may be particularly important when data from a multi-study consortium will be harmonized or pooled for analysis. These functions could be fulfilled by investigators from other centers, by a coordinating center, or by independent implementation scientists who are knowledgeable about the study design and settings.

### Challenges and future directions

Challenges we surmounted in the development and piloting of LISTS within our multicenter consortium included balancing precision, accuracy, and comprehensiveness of reporting, achieving a reporting frequency interval that minimized the biases of retrospection, addressing staff burden, and providing training to build each RC’s capacity to efficiently and effectively use the LISTS method. Evaluation data indicate the need for streamlining procedures to reduce burden and improve the usability of the LISTS data capture interface. Relatedly, the incremental value of capturing very specific and detailed data on implementation strategies for the purpose of complete reporting and specification of strategies [8], relative to existing approaches that capture fewer data elements and with much less specificity [13], needs to be demonstrated.

The use of CFIR and ERIC offered some advantages (i.e., uniformity in terminology and building off taxonomies that are well-established and familiar to implementation scientists) but also some disadvantages (i.e., lack of clear delineations between certain determinants/strategies and terminology that required dedicated involvement of an implementation scientist within each team). LISTS could be used with other frameworks, models, and strategy taxonomies, such as those that are specific to particular types of interventions and/or contexts [14, 28, 29]. Use of alternative, new, or updated frameworks and strategy taxonomies (e.g., CFIR 2.0 [30]) will necessitate updating the data capture platform.

Key next steps for strengthening the LISTS method and encouraging its continued adoption include 1) testing LISTS in other implementation studies; 2) refinement of the LISTS User’s Guide to reduce time for data entry and classification; 3) augmentation of LISTS data with qualitative data (e.g., in elaborating the process of and reasons for modifying strategies); 4) adaptation to different types of studies, including versions for different designs and single versus multi-study use; 5) development of different versions of the data capture platform to increase scalability (e.g., a web-based interface will soon be hosted at https://hivimpsci.northwestern.edu/tools/), as potential users may not have institutional access to REDCap; and 6) other considerations such as capacity building in settings with limited implementation science expertise.

Lastly, into the future, LISTS could also incorporate additional modules to collect data on additional aspects described in FRAME-IS (content, process, frequency, and for what purpose modifications occurred) and data to aid in time-based activity-driven strategy costing methods [31] given that actors and time (dose and duration/temporality) are captured for each strategy in LISTS. Data output of LISTS is also an area of future development. Smith et al. [32] provide an example of a timeline-based figure of when strategies captured in LISTS were used over the course of an implementation project independent of, but with similar aims to, those of the IMPACT Consortium (i.e., implementation of PROs to track cancer symptoms [33]). Different strategy data visualization and output formats could improve the utility of strategy tracking.

### Recommendations for using LISTS

Based on data presented herein, we provide some initial recommendations for research teams to consider in deciding whether or not to use the LISTS method in their own studies. While developed as being as pragmatic as possible, the LISTS method still necessitates monthly or quarterly meetings of multiple team members, as well as additional time between meetings, to adequately track required strategy specification and modification data. Team composition should include a mix of practitioners or implementers, operational leaders, healthcare providers, and program managers to provide a broad and complementary perspective on strategy use. Including an implementation scientist is also recommended to aid in identifying and categorizing implementation strategies based on the ERIC compilation, for which non-implementation scientists may be less familiar. Users will also need to plan for the resources required in the project budgeting and staffing process.

This also raises the questions of when *could* and when *should* teams invest the resources to use LISTS. LISTS could be used for any implementation research project where one or more strategy is being deployed and evaluated. Some indications for when LISTS should be used (i.e., when it is particularly well-suited for a study) concern duration of the strategy use (i.e., more appropriate for longer duration studies), potential for change over time (e.g., if there is higher likelihood of changes due to flexibility afforded to implementers or simply less control over what implementers do), and the degree to which consistency (or understanding and documenting variation) is critical to the research design. For example, it might be more important to demonstrate fidelity to the implementation strategy in a randomized implementation trial than in an observational study. Similarly, in stepped-wedge and other roll-out implementation trial designs, which all RCs in the IMPACT Consortium were, documenting differences between clusters and sites/clinics within clusters is critical to internal validity and interpretation of the findings. Similarly, LISTS can also be useful in tracking protocol deviations in implementation trials to aid in understanding any differences that may occur within and between study conditions over time.

## Conclusions

The newly developed LISTS method is a systematic, comprehensive, and standardized approach for tracking implementation strategies and their modification across time. The components, content, and structure of LISTS build upon existing strategy tracking methods reported in the literature while also addressing some of their limitations. Although initial evaluation data indicates that LISTS is generally feasible, acceptable, and easy to use, our evaluation findings suggest that there are opportunities for improvement, particularly with respect to how to balance rigor and precision around strategy specification across time, the need for inclusion of implementation science expertise to ensure reliable characterization of strategies and determinants according to implementation science taxonomies and frameworks (e.g., ERIC, CFIR), and the potential burdens of data entry and data cleaning and validation.

Overall, the LISTS method was developed as part of a broader effort in the field to improve our empirical understanding of what strategies are used in various contexts, and how and why they are sustained, modified, or discontinued over time. LISTS provides a systematic approach for strategy assessment, data capture, and procedures to facilitate a curated central repository with common data elements. Initial use and evaluation of the LISTS method in three ongoing hybrid effectiveness-implementation studies reveals LISTS as a promising approach for measuring and reporting implementation strategies over time. Future research is needed to further evaluate, improve, and adapt components of LISTS to fit the context of different research studies and distinct implementation science theories, models, and frameworks, thereby expanding our empirical understanding of implementation strategies.

### Supplementary Information

**Additional file 1.**

**Additional file 2.**

**Additional file 3.**

## Data Availability

Data presented in this article are not available given they cannot be deidentified. The LISTS REDCap program is maintained by the Dissemination and Implementation Science Core of the University of Utah Clinical and Translational Science Institute and is available on GitHub at https://github.com/uofu-ccts/LISTS_REDCap_project.

## Abbreviations

CFIR: Consolidated Framework for Implementation Research
EHR: Electronic Health Record
ERIC: Expert Recommendations for Implementing Change
FRAME: Framework for Reporting Adaptations and Modifications
FRAME-IS: Framework for Reporting Adaptations and Modifications to Evidence-based Implementation Strategies
IMPACT: Improving the Management of sympToms during And following Cancer Treatment
LISTS: Longitudinal Implementation Strategy Tracking System
NCI: National Cancer Institute
RC: Research Center
RE-AIM: Reach, Effectiveness, Adoption, Implementation, and Maintenance
REDCap: Research Electronic Data Capture
SUS: System Usability Survey
