# Supplementary material for: The Longitudinal Implementation Strategy Tracking System (LISTS): feasibility, usability, and pilot testing of a novel method
Source: Implement Sci Commun. 2023 Nov 28;4:153. doi: 10.1186/s43058-023-00529-w (PMC10683230; doi:10.1186/s43058-023-00529-w)
Supplement: Supplementary file 1 — Additional file 1. [file 43058_2023_529_MOESM1_ESM.docx]

IMPACT Consortium Members

| **Full Name and Degrees** | **Institution** | **Email** |
| --- | --- | --- |
| Deborah Schrag, MD, MPH | Memorial Sloan Kettering Cancer Institute, New York, NY | schragd@mskcc.org |
| Sandra L. Wong, MD, MD | Dartmouth-Hitchcock Medical Center, Lebanon, NH | Sandra.L.Wong@Hitchcock.org |
| Barbara L. Kroner, PhD, MPH | RTI International, Research Triangle Park, NC | [byk@rti.org](mailto:byk@rti.org) |
| Ashley Wilder Smith, PhD, MPH | National Cancer Institute, Bethesda, MD | [smithas@mail.nih.gov](mailto:smithas@mail.nih.gov) |
| Joan Griffin, PhD | Mayo Clinic, Rochester MN | [griffin.joan@mayo.edu](mailto:griffin.joan@mayo.edu) |
| Roxanne Jensen, PhD | National Cancer Institute, Bethesda, MD | [roxanne.jensen@nih.gov](mailto:roxanne.jensen@nih.gov) |
| Kathryn Ruddy, MD, MPH | Mayo Clinic, Rochester MN | [ruddy.kathryn@mayo.edu](mailto:ruddy.kathryn@mayo.edu) |
| Betina Yanez, PhD | Northwestern University Feinberg School of Medicine, Chicago, IL | [betina.yanez@northwestern.edu](mailto:betina.yanez@northwestern.edu) |
| Jessica J. Bian, MD | Maine Medical Center, Portland, ME | Jessica.Bian@mainehealth.org |
| Hannah W. Hazard-Jenkins, MD, FACS | West Virginia University Cancer Institute, Morgantown, WV | [hhazard@hsc.wvu.edu](mailto:hhazard@hsc.wvu.edu) |
| Mary-Anne Ardini | RTI International, Research Triangle Park, NC | [maa@rti.org](mailto:maa@rti.org) |
| Paige Ahrens, MS | Maine Medical Center, Portland, ME | [Paige.Ahrens@mainehealth.org](mailto:Paige.Ahrens@mainehealth.org) |
| Fiona Barrett | Dana-Farber Cancer Institute, Boston, MA | [fionag_barrett@dfci.harvard.edu](mailto:fionag_barrett@dfci.harvard.edu) |
| Michael Bass, MS | Northwestern University Feinberg School of Medicine, Chicago, IL | [michael-bass@northwestern.edu](mailto:michael-bass@northwestern.edu) |
| Megan Begnoche, RN, MSN | Lifespan Cancer Institute, Providence, RI | [mbegnoche@lifespan.org](mailto:mbegnoche@lifespan.org) |
| Kimberly Caron, RN, BSN, CCRC | Maine Medical Center, Portland, ME | [Kimberly.Caron@mainehealth.org](mailto:Kimberly.Caron@mainehealth.org) |
| Linda Chlan, PhD, RN | Mayo Clinic, Rochester MN | [Chlan.Linda@mayo.edu](mailto:Chlan.Linda@mayo.edu) |
| Ava Coughlin, MAEd | Northwestern University Feinberg School of Medicine, Chicago, IL | [ava.coughlin@northwestern.edu](mailto:ava.coughlin@northwestern.edu) |
| Samira Dias, MPH | Dana-Farber Cancer Institute, Boston, MA | samiral_dias@dfci.harvard.edu |
| Nicolas Faris, M.Div | Baptist Memorial Hospital, Memphis, TN | [Nick.Faris@BMHCC.org](mailto:Nick.Faris@BMHCC.org) |
| Ann Marie Flores, PhD, PT | Northwestern University Feinberg School of Medicine, Chicago, IL | [ann.flores@northwestern.edu](mailto:ann.flores@northwestern.edu) |
| Martha Garcia | Northwestern University Feinberg School of Medicine, Chicago, IL | [martha.garcia@northwestern.edu](mailto:martha.garcia@northwestern.edu) |
| Karla Hemming, PhD | University of Birmingham, Edgbaston, Birmingham, UK | [k.hemming@bham.ac.uk](mailto:k.hemming@bham.ac.uk) |
| Jeph Herrin, PhD, MS | Yale University School of Medicine, New Haven, CT | [jeph.herrin@yale.edu](mailto:jeph.herrin@yale.edu) |
| Christine Hodgdon, MS | GRASP, Baltimore, MD | [chodgdon513@gmail.com](mailto:chodgdon513@gmail.com) |
| Sheetal Kircher, MD | Northwestern University Feinberg School of Medicine, Chicago, IL | [sheetal.kircher@nm.org](mailto:sheetal.kircher@nm.org) |
| Kurt Kroenke, MD, MAC | Indiana University, Indianapolis, IN | [kkroenke@regenstrief.org](mailto:kkroenke@regenstrief.org) |
| Veronica Lam | Mayo Clinic, Rochester MN | [Lam.Veronica@mayo.edu](mailto:Lam.Veronica@mayo.edu) |
| Nicola Lancki, MPH | Northwestern University Feinberg School of Medicine, Chicago, IL | [nicola.lancki1@northwestern.edu](mailto:nicola.lancki1@northwestern.edu) |
| Quan H. Mai, MS | Northwestern University Feinberg School of Medicine, Chicago, IL | [quan.mai@northwestern.edu](mailto:quan.mai@northwestern.edu) |
| Jennifer Mallow, PhD, FNP-BC | West Virginia University Cancer Institute, Morgantown, WV | [jamallow@hsc.wvu.edu](mailto:jamallow@hsc.wvu.edu) |
| Nadine J. McCleary, MD, MPH | Dana-Farber Cancer Institute, Boston, MA | [nj_mccleary@dfci.harvard.edu](mailto:nj_mccleary@dfci.harvard.edu) |
| Mary O'Connor, MS | Northwestern University Feinberg School of Medicine, Chicago, IL | [mary.oconnor@northwestern.edu](mailto:mary.oconnor@northwestern.edu) |
| Deirdre Pachman, MD | Mayo Clinic, Rochester MN | [Pachman.Deirdre@mayo.edu](mailto:Pachman.Deirdre@mayo.edu) |
| Loretta Pearson, Mphil, CCRC | Dartmouth-Hitchcock Medical Center, Lebanon, NH | [Loretta.H.Pearson@Hitchcock.org](mailto:Loretta.H.Pearson@Hitchcock.org) |
| Frank Penedo, PhD | University of Miami, Miami FL | [fpenedo@miami.edu](mailto:fpenedo@miami.edu) |
| Jewel Podratz, MBA | Mayo Clinic, Rochester MN | [podrj@mayo.edu](mailto:podrj@mayo.edu) |
| Jennifer Popovic, DVM, MA | RTI International, Research Triangle Park, NC | [jennifer.popovic@gmail.com](mailto:jennifer.popovic@gmail.com) |
| Liliana Preiss, MSE | RTI International, Research Triangle Park, NC | [preiss@rti.org](mailto:preiss@rti.org) |
| Parvez Rahman, MHI | Mayo Clinic, Rochester MN | [Rahman.Parvez@mayo.edu](mailto:Rahman.Parvez@mayo.edu) |
| Sarah Minteer, PhD, MA | Mayo Clinic, Rochester MN | [Minteer.Sarah@mayo.edu](mailto:Minteer.Sarah@mayo.edu) |
| James Reich, PMP | Maine Medical Center, Portland, ME | James.Reich@mainehealth.org |
| Kimberly Richardson, MA | Black Cancer Collaborative, Chicago, IL | [kdrichardson0924@gmail.com](mailto:kdrichardson0924@gmail.com) |
| Lila Rutten, PhD | Mayo Clinic, Rochester MN | [rutten.lila@mayo.edu](mailto:rutten.lila@mayo.edu) |
| Karen Schaepe, PhD | Mayo Clinic, Rochester MN | [Schaepe.Karen@mayo.edu](mailto:Schaepe.Karen@mayo.edu) |
| Denise Scholtens, PhD | Northwestern University Feinberg School of Medicine, Chicago, IL | [dscholtens@northwestern.edu](mailto:dscholtens@northwestern.edu) |
| Tiana Poirier-Shelton, MPH | Baptist Memorial Hospital, Memphis, TN | [tiana.shelton@bmhcc.org](mailto:tiana.shelton@bmhcc.org) |
| Philip Silberman, MA | Northwestern University Feinberg School of Medicine, Chicago, IL | [psilber1@nm.org](mailto:psilber1@nm.org) |
| Jaclyn Simpson, MBA | Baptist Memorial Hospital, Memphis, TN | Jaclyn.Simpson@bmg.md |
| Laura Tasker, BS, RT(N) | West Virginia University Cancer Institute, Morgantown, WV | [LATASKER@hsc.wvu.edu](mailto:LATASKER@hsc.wvu.edu) |
| Cindy Tofthagen, PhD | Mayo Clinic, Jacksonville FL | [Tofthagen.Cindy@mayo.edu](mailto:Tofthagen.Cindy@mayo.edu) |
| Angela Tramontano, MPH | Dana Farber Cancer Institute, Boston, MA | [angela_tramontano@dfci.harvard.edu](mailto:angela_tramontano@dfci.harvard.edu) |
| Benjamin D. Tyndall, PhD | RTI International, Research Triangle Park, NC | [btyndall@rti.org](mailto:btyndall@rti.org) |
| Hajime Uno, PhD | Dana-Farber Cancer Institute, Boston, MA | [huno@ds.dfci.harvard.edu](mailto:huno@ds.dfci.harvard.edu) |
| Kimberly Webster, MA | Northwestern University Feinberg School of Medicine, Chicago, IL | [k-webster@northwestern.edu](mailto:k-webster@northwestern.edu) |
| Bryan Weiner, PhD, MA | University of Washington, Seattle, WA | [bjweiner@uw.edu](mailto:bjweiner@uw.edu) |
