# Supplementary material for: The Longitudinal Implementation Strategy Tracking System (LISTS): feasibility, usability, and pilot testing of a novel method
Source: Implement Sci Commun. 2023 Nov 28;4:153. doi: 10.1186/s43058-023-00529-w (PMC10683230; doi:10.1186/s43058-023-00529-w)

**Supplemental File 1**

**Screenshots of REDCap Data Entry Tool**


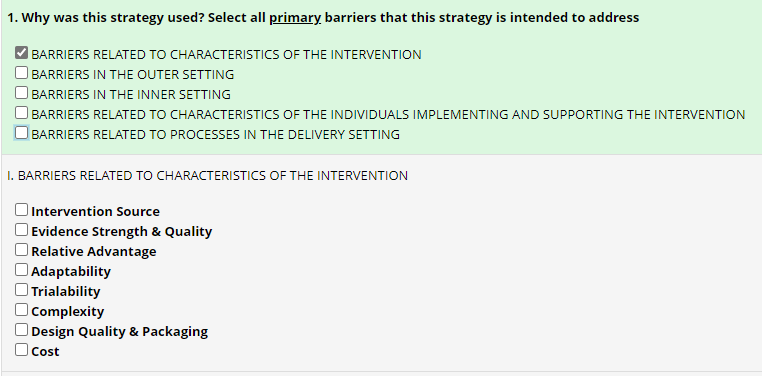
Supplemental Figure 1. Example of Implementation Barriers from the Characteristics of the Intervention Domain


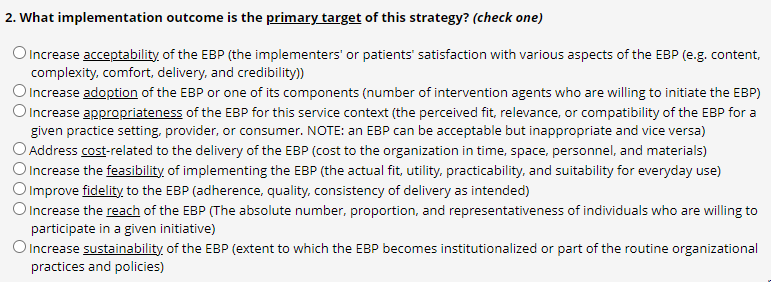
 Supplemental Figure 2. Implementation Outcomes


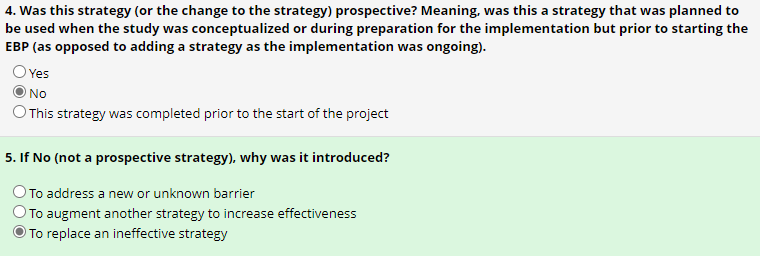
 Supplemental Figure 3. Unplanned Strategy Modification

Supplemental Figure 4. Specifying Study Units


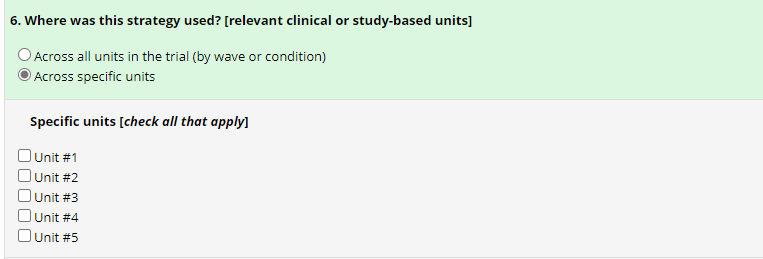


Supplemental Figure 5. Record Status Dashboard


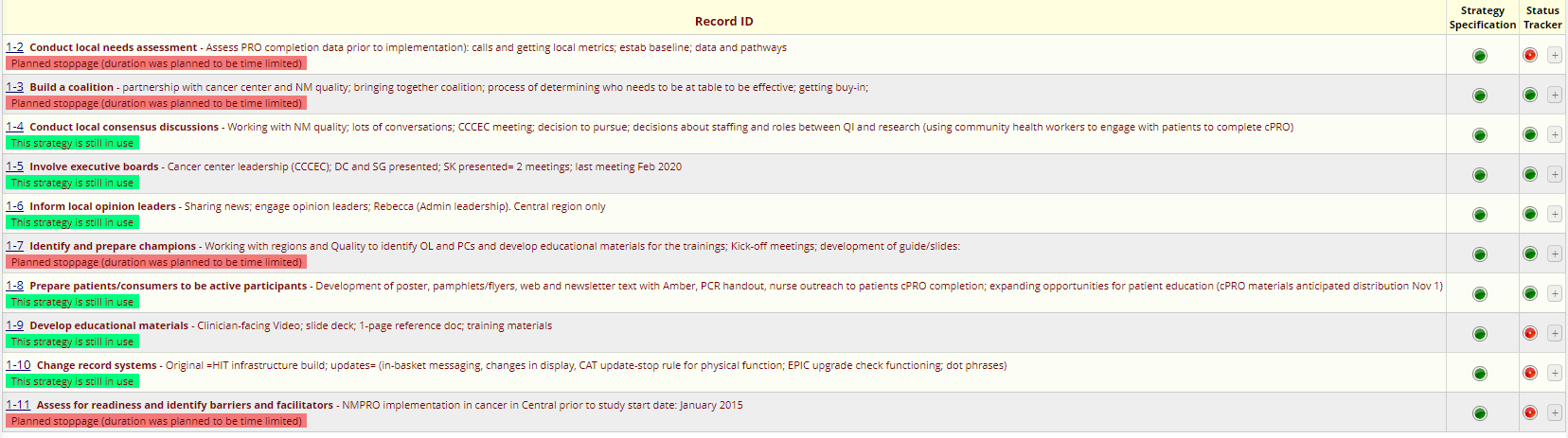

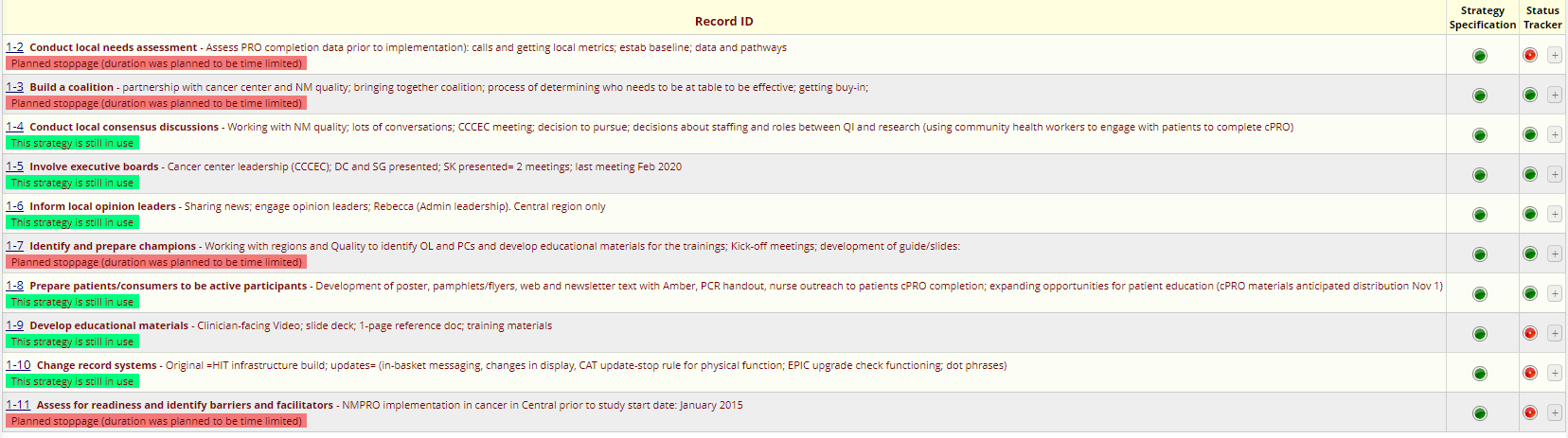


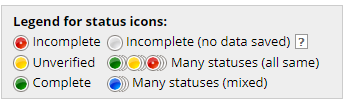

Supplement: Supplementary file 2 — Additional file 2. [file 43058_2023_529_MOESM2_ESM.docx]
