# Supplementary material for: The Longitudinal Implementation Strategy Tracking System (LISTS): feasibility, usability, and pilot testing of a novel method
Source: Implement Sci Commun. 2023 Nov 28;4:153. doi: 10.1186/s43058-023-00529-w (PMC10683230; doi:10.1186/s43058-023-00529-w)
Supplement: Supplementary file 3 — Additional file 3. [file 43058_2023_529_MOESM3_ESM.docx]

**Supplemental File 2**

**LISTS Feasibility and Usability Data Collection Form**

*During the next meeting with your implementation strategies team, please provide the following information about your team’s use of LISTS (Longitudinal Implementation Strategies Tracking System). Responses may be based on discussion, notes, and any other information you may have to help facilitate recall and answer the questions to the best of your ability. Please feel free to add more rows in the response tables as needed.*

IMPACT Research Center:

1. List the dates your RC team has met to discuss LISTS. For each meeting, please indicate the amount of time devoted to: ***initial population*** of *strategies* [entry of strategies not previously reported] and ***updating*** *already entered strategies* [reporting on modifications/stoppages/etc.]

|  |  | Time (min) | |
| --- | --- | --- | --- |
|  | **Date** (Day/Month/Year or Month/Year) | **Initial population** | **Updating** |
| 1. |  |  |  |
| 2. |  |  |  |
| 3. |  |  |  |
| 4. |  |  |  |
| 5. |  |  |  |
| 6. |  |  |  |

1. Describe the role of the individuals who have comprised the group completing LISTS (e.g., project coordinator, implementation scientist, quality improvement leads, physicians, nurse manager, etc.). Indicate whether this person has been involved in meetings routinely (majority of meetings) or occasionally (minority of meetings):

|  | Role description (name optional) | **Routine** or **Occasional** (choose one) |
| --- | --- | --- |
| a. |  |  |
| b. |  |  |
| c. |  |  |
| d. |  |  |

1. Describe the process your RC used for the **initial population** of LISTS. Such as went through all ERIC strategies to assess whether they were used, started from the protocol/research proposal, used Excel spreadsheets, emailed implementers/Actors of the strategies, etc.

|  |
| --- |

1. Describe the process for **updating** of LISTS (i.e., any data entries that occurred after the initial assessment was completed). Such as, emailing implementers/Actors of the strategies for updates, etc.

|  |
| --- |

1. Which of the following methods do you use to complement and/or confirm the accuracy of data entered in LISTS? Indicate use and, if used, the frequency.

|  | **Use**  [enter an “X” if used ≥once | **Frequency**  1=Rarely (once or twice)  2=Occasionally (a few times)  3=Frequently (many times)  4=Always (nearly every strategy) |
| --- | --- | --- |
| Review of meeting notes/agendas (e.g., for topics discussed, persons involved) |  |  |
| Review of calendar entries (e.g., for meeting dates) |  |  |
| Checking with on-the-ground staff/ implementers (e.g., for Actor, Action, time involved) |  |  |

1. Rate the difficulty of assessing each of the following elements of LISTS.

| **LISTS Element** | **Difficulty**  1 = Very Easy  2 = Easy  3 = Neutral (neither difficult or easy)  4 = Difficult  5 = Very Difficult |
| --- | --- |
| Selecting a strategy category (from ERIC compilation) |  |
| Selecting a specific strategy (from ERIC compilation) |  |
| Reason for strategy stoppage |  |
| Why was strategy used (i.e., selection of barriers strategy thought to be addressing) |  |
| Implementation outcome target |  |
| Prospective vs. not |  |
| Location of strategy use |  |
| Actor(s) who used the strategy |  |
| Frequency of strategy use |  |
| How long does it take to do the strategy (dose) |  |
| Individuals involved in the strategy |  |

1. Rate your team’s reactions to the LISTS data acquisition form in REDCap as of *today*.

|  | 1 = Strongly Disagree  2 = Disagree  3 = Neither Agree or Disagree  4 = Agree  5 = Strongly Agree |
| --- | --- |
| I think that I would like to use the LISTS REDCap system frequently. |  |
| I found the LISTS REDCap system unnecessarily complex. |  |
| I thought the LISTS REDCap system was easy to use. |  |
| I think that I would need assistance to be able to use the LISTS REDCap system. |  |
| I found the various functions/questions in the LISTS REDCap system were well integrated. |  |
| I thought there was too much inconsistency in the LISTS REDCap system. |  |
| I would imagine that most people would learn to use the LISTS REDCap system very quickly. |  |
| I found the LISTS REDCap system very cumbersome/awkward to use. |  |
| I felt very confident using the LISTS REDCap system. |  |
| I needed to learn a lot of things before I could get going with the LISTS REDCap system. |  |

1. Please provide any comments about the LISTS REDCap form.

|  |
| --- |

1. Is there any other information you would like to provide about LISTS to help understand how it is being used by your RC?

|  |
| --- |
